# Second to fourth (2D:4D) digit ratio and their relationships among a mother and child population in Ghana

**DOI:** 10.1038/s41598-021-92358-x

**Published:** 2021-06-22

**Authors:** Moses Banyeh, Nafiu Amidu, Lawrence Quaye

**Affiliations:** Department of Biomedical Laboratory Science, School of Allied Health Sciences, University for Development Studies, Tamale, Ghana

**Keywords:** Biochemistry, Developmental biology, Anatomy

## Abstract

The study aimed to determine the relationship between digit ratios among a mother–child population in Ghana. This was a cross-sectional study from December 2020 to April 2021 involving 272 mothers, their daughters (n = 132) and their sons (n = 140). The right (2D:4DR) and the left (2D:4DL) digit ratios were measured using computer-assisted analysis. The data were analysed in SPSS (v23) and GraphPad Prism (v8) at an alpha value of 0.05. The mean ± SD age of the mothers was 23.9 ± 3.67 years while the median (IQR) age of daughters was 116(54–240) days and sons, 134(54–240) days. The mean ± SD 2D:4DR were 0.94 ± 0.04, 0.91 ± 0.04 and 0.90 ± 0.04 respectively for mothers, daughters and sons. The mean ± SD 2D:4DL was 0.93 ± 0.04, for mothers, 0.92 ± 0.05 for daughters and 0.92 ± 0.05 for sons. The daughters and sons showed leftward asymmetry while the mothers showed rightward asymmetry in digit ratios. The 2D:4DR of sons was significantly lower than daughters (P = 0.031). There were negative correlations between the 2D:4DL and age of daughters (r = −0.182, P = 0.043) and sons (r = −0.221, P = 0.012). The 2D:4DR of mothers was positively correlated with that of daughters (r = 0.332, P = 0.000) and that of sons (r = 0.233, P = 0.008). There are significant relationships between digit ratios in a mother–child population.

## Introduction

The second to fourth digit (2D:4D) ratio is a putative biomarker of prenatal testosterone and oestrogen exposure^1,2^. According to the “Organizational hypothesis”, prenatal androgen (testosterone) exposure has a permanent masculinizing effect on brain organization and personality leading to sexually dimorphic traits. Prenatal androgen exposure has been suggested to be inversely correlated to the 2D:4D digit ratio^3,4^. It is an easy, simple and non-invasive alternative method to amniocentesis in the investigation of in utero effect of androgen activity on human digit development^5^.

Previous studies have sought to investigate the sexually dimorphic nature of the 2D:4D ratio but with varied outcomes. While some studies found reduced digit ratios in males as compared to females, others did not^6,7^. The association between age and digit ratios have also been studied. While Manning, et al.^8^ earlier hypothesized that the 2D:4D ratio fluctuates and only gains stability after 2 years of age, other longitudinal and population studies have found that the 2D:4D digit ratios increased with age^4,9,10^. Also, studies have shown that directional asymmetry which is defined as the right-left difference in digit ratio (Dr-l) are frequently leftward in males and rightward in females^11^.

The relationship between digit ratios in mother–child pairs have been demonstrated by previous authors including Shere, et al.^12^ and Kalichman, et al.^13^ who found significant correlations in mother–child digit ratios. Previous familial studies on digit ratios have indicated that the heritability of the 2D:4D digit ratio was up to 57% for the right hand and 48% for the left -hand^14^. Although the process of digit ratio heritability is not well understood and will require further studies, genetic factors have been suggested^12,14^.

The 2D:4D ratio shows genetic and environmental variability such that findings from one population may not be extrapolated to another population. There is therefore the need for population-specific studies of digit ratios and their relationships. This study, therefore, aimed to determine the 2D:4D ratios and how they are related in a mother–child population in Ghana.

## Methods

### Study design and population

This was a cross-sectional study from December 2020 to April 2021. The study recruited first-time mothers, and their children, who were receiving postnatal services at the Reproductive and Child Health (RCH) clinic, located in the Tamale Metropolis in the Northern region of Ghana. The RCH is well patronized by young mothers and was therefore suitable for mother–child pair studies^15^. The inclusion and exclusion criteria were; a first-time mother with singleton birth ≤ 730 days (≤ 2 years) old. All participants were to be devoid of limb, finger and spinal deformities and also without any known medical history of congenital adrenal hyperplasia (CAH). The authors, however, could not have excluded participants with a previous history of fractures that had healed and not apparent to the observers. This was because X-ray images were not available.

### Data collection and anthropometric measurements

Socio-demographic data such as age, ethnicity, religion, marital status, employment status, and educational level were collected through a structured interview and questionnaire. The right and left palmar surfaces of the hands of all the mothers and their children were scanned following the technique by Neyse and Brañas-Garza^16^. Participants were asked to remove all objects including rings that could mask creases or reflect light during the scanning process. The mother was then guided to place the ventral palmar surface of each hand on the flatbed surface of an HP desk jet 2620 all-in-one printer scanner (HP Inc. 1501 Page Mill Road Palo Alto, CA 94304 United States). The mother was asked to press the hand firmly enough making the fingers straight and visible to obtain a good scan but not push the glass with the fingertips. The mother was told not to move her hand during the scanning process. The second to fifth fingers were held parallel and the tip of the middle finger aligned with the wrist and elbow (Allaway, Bloski, Pierson, & Lujan, 2009). A unique identification number, e.g., LH-001M for the left hand of the first mother or LH-001C for the left hand of her child was boldly written on a piece of paper and placed on the flatbed of the scanner alongside the hand. When scanning the right hand, ‘RH’ was used in place of ‘LH’. The hands were then scanned at a resolution of 150 dpi. In the case of the children, one observer had to hold the hands and place them as appropriate on the scanner while the second observer issued the command to scan the hand. The scanned images were then stored with the ID number on a laptop for further analysis. The scanned images were later exported into GIMP (v 2.10.22), an image manipulation program (www.gimp.org) for digit measurements. The resolution of the image was adjusted as appropriate to ensure the proper delineation and visibility of creases. The calliper in GIMP was controlled with the mouse pointer. The pointer was placed on the proximal crease and then extend to the tip of the finger. The length was adjusted as appropriate before the results were read from the results window to the nearest 0.01 mm. The process was repeated by the same observer after 1 week. The digit ratio was calculated by dividing the 2D by the 4D for the first and second measurements and the 2 were then averaged to obtain the final results. The reliability of the repeat measurements was assessed by calculating the intraclass correlation coefficients (two-way mixed, single measures with absolute agreement). The 2D:4DL had an ICC of r = 0.996 (95%CI 0.994–0.998), while the right 2D:4D ratio ICC was r = 0.984 (95%CI 0.980–0.989), The other anthropometric variables; weight and height were measured following the recommendations of Best and Shepherd^17^ using a stadiometer for height and a bathroom scale for body weight. All measurements were carried out between 08.00 − 12.00 h GMT to avoid diurnal variations in measurements.

### Statistical analysis

The data were entered into a Microsoft Excel spreadsheet and then exported to SPSS (v23) and GraphPad Prism (v 8) statistical software for analysis. Descriptive statistics were performed for each variable and differences between means or medians among daughters and sons were determined using a student *t*-test (unpaired, 2-tailed) for parametric and the Mann–Whitney U test (unpaired, 2-tailed) for non-parametric variables. To assess differences between the left and right digit ratios within each group (mothers, daughters and sons), the paired student *t*-test (2-tailed) was used. The relationships between digit ratios and age, digit ratios of mothers and their daughters and mothers and their sons were performed using Pearson correlation. To determine the differences in relationships between age and the left and right digit ratios, the digit ratios of both hands were regressed on age using linear regression. Statistical significance was considered at an alpha value of 0.05.

### Ethics declarations

All methods were carried out following the relevant international, national and institutional guidelines and regulations. The study received approval from the Institutional Review Board of the University for Development Studies, Tamale. Informed consent was obtained from all the mothers and also, informed parental consent was obtained for each child.

## Results

### Sociodemographic and anthropometric characteristics

**Figure 1:**
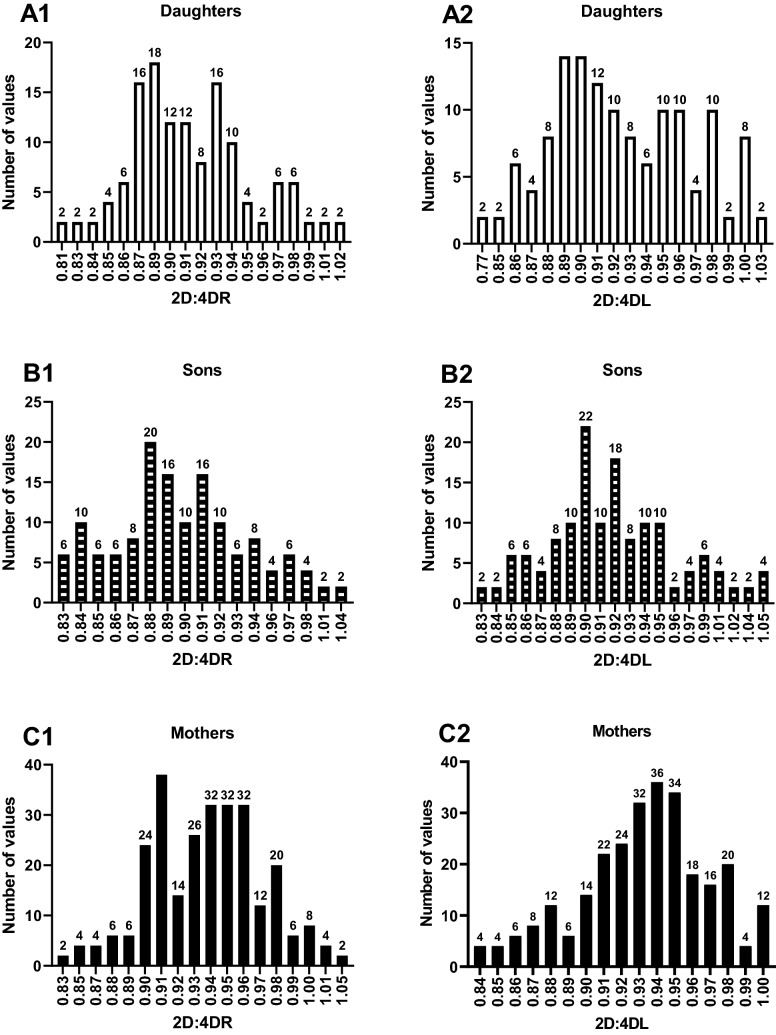
Frequency distribution bar graphs of digit ratios: distribution of digit ratios among daughters (**A1** = 2D:4DR, **A2** = 2D:4DL), distribution of digit ratios among sons (**B1** = 2D:4DR, **B2** = 2D:4DL), distribution of digit ratios among mothers (**C1** = 2D:4DR, **C2** = 2D:4DL).

The sociodemographic and anthropometric characteristics of the mother and child population are summarized in Fig. 1 and Tables 1, 2, 3 and 4. The mothers were aged between 18 and 36 years with a mean ± SD age of 23.9 ± 3.67 years. The 2D:4DR ranged from 0.83 to 1.05 with a mean ± SD of 0.94 ± 0.04, while the 2D:4DL ranged from 0.84 to 1.00 with a mean ± SD of 0.93 ± 0. 04. The 2D:4DR was significantly higher than the 2D:4DL (P = 0.020). The majority [51.5% (140/272)] of the mothers had sons, while the rest had daughters (Table 2).

**Table 1:** Sociodemographic and anthropometric variables of the daughters and the sons involved in the study.

Variable	Daughters n (132)	Sons n (140)	P-value
Age (days)	116 (51–240)	134 (51–240)	0.808
2D:4DR	0.91 ± 0.04‡	0.90 ± 0.04†	0.031
2D:4DL	0.92 ± 0.05	0.92 ± 0.05	0.555
Dr-l	− 0.013 ± 0.048	− 0.021 ± 0.052	0.193

Results presented as mean ± S D for parametric or median (IQR) for non-parametric variables. The mean or median values of sons were compared with those of daughters using the student *t*-test (unpaired, 2-tailed) for parametric and the Mann-witney U test (unpaired, 2-tailed) for non-parametric variables. Also, an intra-sex comparison of the right and the left digit ratio was performed using a student *t*-test (paired, 2-tailed).

^‡^Significant at P = 0.002 when compared to 2D:4DL.

^†^Significant at P < 0.001 when compared to 2D:4DL.

**Table 2:** Sociodemographic (continuous) and anthropometric variables of the mothers involved in the study.

Variable	Minimum	Mean	SD	Maximum
Age (years)	18.0	23.9	3.67	36.0
Height (cm)	145	162	5.63	176
Weight (kg)	45.3	61.9	9.44	91.5
BMI (kg/m^2^)	17.3	23.6	3.56	34.8
2D:4DR	0.83	0.94	0.04	1.05
2D:4DL	0.84	0.93*	0.04	1.00
Dr-l	− 0.070	0.004	0.031	0.080

Results of socio-demographic characteristics (continuous) and anthropometric variables of the 272 mothers presented as minimum, mean, standard deviation (SD) and maximum values for each variable.

*Significant at P = 0.020 when compared to the 2D:4DR (paired *t*-test, 2-tailed).

**Table 3:** Sociodemographic variables (categorical) of the mothers involved in the study.

Variable	n (%)
Number of mothers	272 (100)
**Ethnicity**	
Mole-Dagomba	260 (95.6)
Other-ethnicity	12 (4.4)
**Religion**	
Islam	240 (88.2)
Christianity	32 (11.8)
**Marital status**	
Married	258 (94.9)
Other partnership	14 (5.1)
**Educational status**	
None/basic	44 (16.2)
JHS	48 (17.6)
SHS/Vocational	118 (43.4)
Tertiary	62 (22.8)
**Employment status**	
Unemployed	152 (55.9)
Self-employed	84 (30.9)
Salaried work	36 (13.3)
**Sex of child**	
Female	132 (48.5)
Male	140 (51.5)

Results of socio-demographic characteristics for categorical variables of the 272 mothers presented as number (per cent).

The median (IQR) age of daughters was 116 (51–240) days while that of sons was 134 (54–240) days. The 2D:4DR of daughters ranged from 0.81 to 1.02 with a mean ± SD of 0.91 ± 0.04 while that of the sons ranged from 0.83 to 1.04 with a mean ± SD of 0.90 ± 0.04. The 2D:4DL of daughters ranged from 0.77 to 1.03 with a mean ± SD of 0.92 ± 0.05. The 2D:4DR of sons ranged from 0.83 to 1.05 with a mean ± SD of 0.92 ± 0.05 and was significantly lower than daughters (P = 0.031). Also, the 2D:4DR was significantly reduced in daughters (P = 0.002) and sons (P < 0.001) when compared to their 2D:4DL (Table 3). The 2D:4DR of the mothers was significantly higher than that of both their sons and their daughters (P < 0.001) while the 2D:4DL of the mothers was significantly higher as compared to their daughters (P = 0.048) and their sons (P = 0.039).

**Table 4:** Comparing digit ratios between mothers, their daughters and their sons.

Variable	Mothers n (132)	Daughters n (132)	P-value	Mothers n (140)	Sons n (140)	P-value
2D:4DR	0.94 ± 0.03	0.91 ± 0.04	< 0.001	0.93 ± 0.04	0.90 ± 0.04	< 0.001
2D:4DL	0.93 ± 0.03	0.92 ± 0.05	0.048	0.93 ± 0.04	0.92 ± 0.05	0.039
Dr-l	0.007 ± 0.032	-0.013 ± 0.048	< 0.001	0.002 ± 0.029	-0.021 ± 0.052	< 0.001

Results were presented as mean ± SD. Digit ratios of mothers were compared to their daughters and their sons using student *t*-test (unpaired, 2-tailed).

### Relationship between digit ratios and age

**Figure 2:**
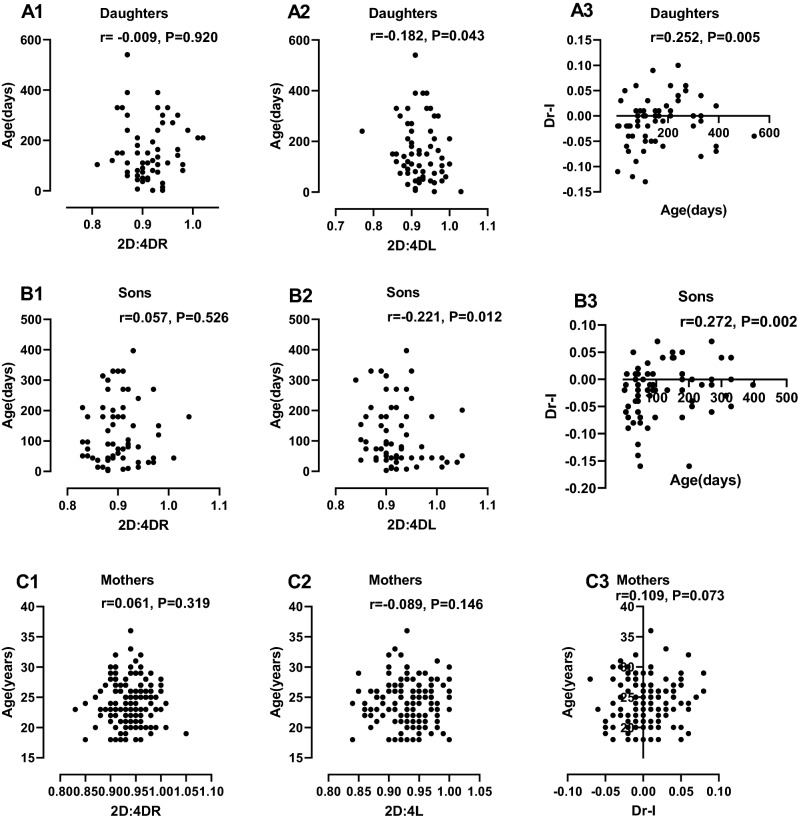
Pearson correlation scatterplots between age and digit ratios: correlation between digit ratios and age among daughters (**A1** = 2D:4DR, **A2** = 2D:4DL, **A3** = Dr-l), correlation between digit ratios and age among sons (**B1** = 2D:4DR, **B2** = 2D:4DL, **B3** = Dr-l), correlation between digit ratios and age among mothers (**C1** = 2D:4DR, **C2** = 2D:4DL, **C3** = Dr-l).

**Figure 3:**
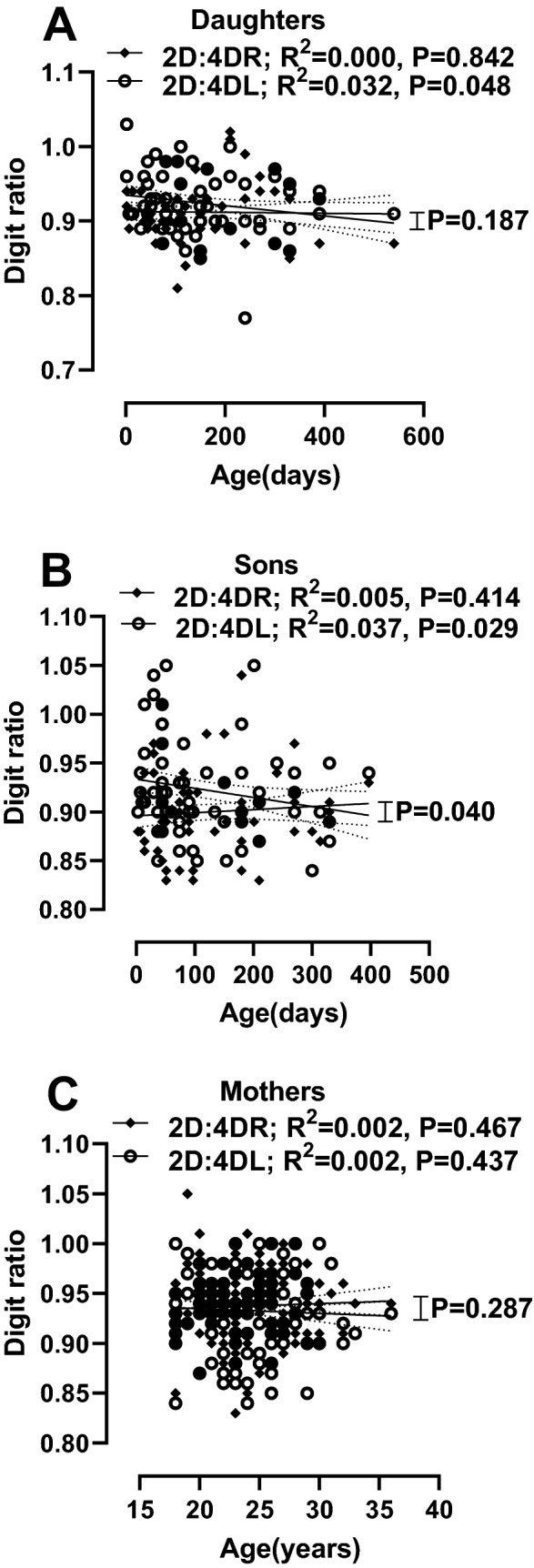
Linear regression plots of right and left digit ratios to age: linear regression of digit ratios to age among daughters (**A**), linear regression of digit ratios to age among sons (**B**), linear regression of digit ratios to age among mothers (**C**).

Pearson correlation was performed between digit ratios and age among the mothers, their daughters and their sons (Fig. 2). There was a significant inverse correlation between 2D:4DL and age of daughters (r = -0.182, P = 0.043). Also, the Dr-l showed a positive and significant relationship with the age of daughters (r = 0.252, P = 0.005). Similarly, the 2D:4DL of the sons was inversely correlated with their age (r = -0.221, P = 0.012). The Dr-l of the sons also showed a positive and significant correlation with age (r = 0.272, P = 0.002). To assess differences in the relationship between the right and the left digit ratios with age, the digit ratios were regressed on the age separately for daughters, sons and their mothers (Fig. 3). The differences in correlation with age between the right and left digit ratios were only significant in sons (P = 0.040).

### Relationship between mother–child digit ratios

**Figure 4:**
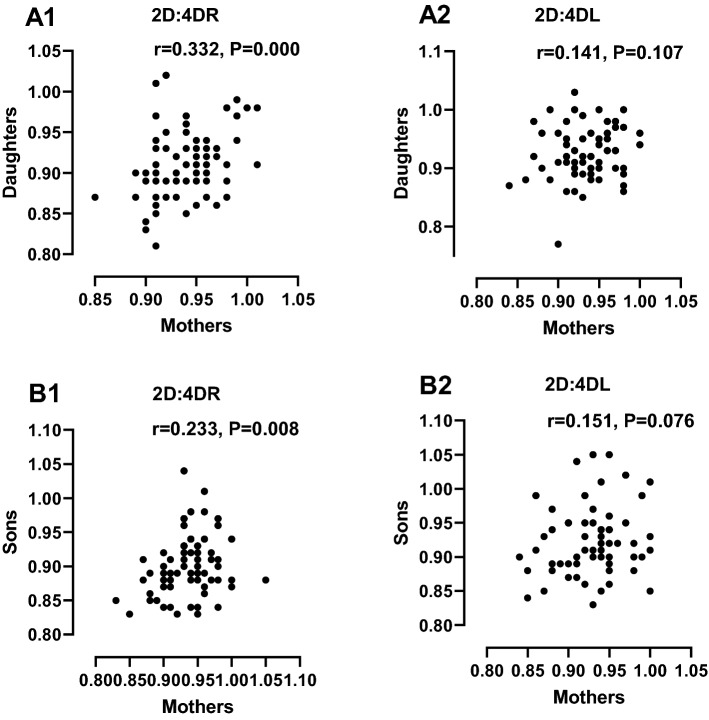
Pearson correlation scatterplots of digit ratios between mothers and their children: correlation of digit ratios between mother and daughter (**A1** = 2D:4DR, **A2** = 2D:4DL), correlation of digit ratios between mother and son (**B1** = 2D:4DR, **B2** = 2D:4DL).

The relationship between digit ratios of mothers and their children was performed using Pearson correlation plots (Fig. 4). The 2D:4DR of the daughters was positively correlated with that of their mothers (r = 0.332, P = 0.000). Also, the 2D:4DR of the sons was positively correlated with that of the mothers (r = 0.223, P = 0.008).

## Discussion

The study aimed to determine 2D:4D ratios and their relationships in the mother–child population in Ghana. It was observed that the mothers showed rightward bias while their sons and daughters showed leftward bias in digit symmetry. Mothers’ digit ratios were significantly higher compared to their sons and daughters. The 2D:4DR in the sons was significantly reduced as compared to that of the daughters. The 2D:4DL in both the sons and daughters were inversely correlated with age. Also, the 2D:4DR of the mothers was positively correlated with the 2D:4DR of their sons and daughters.

According to Manning, et al.^8^, the proof of the effect of prenatal androgen exposure on digit ratio is indicated by the occurrence of sexual dimorphism in digit ratios at an early stage in life. This current study found that the 2D:4DR of sons was significantly reduced compared to daughters. According to Tanner and Tanner^18^, the right digit ratio turned to be “male-like” or reduced in male and this may be due to increased sensitivity of the right-hand digits to prenatal androgen exposure as compared to the left^8^. A previous study by Manning, et al.^19^ found significantly reduced 2D:4DR in boys, compared to girls among Jamaican children aged 5–11 years. In their study, Manning, et al.^19^ measured digit ratios directly and also, indirectly (from photocopies). A previous study by, Ventura, et al.^7^ among 106 newborn babies in a prospective study conducted at the maternity of Dona Estefânia Hospital (HDE) in Lisbon, found reduced 2D:4DL in baby boys. The authors measured digit lengths from photocopies of the ventral palmar surface and observed substantial overlap between male and female babies with only subtle differences. Similar to the study of Ventura, et al.^7^, a study by Ertuğrul, et al.^20^, involving 225 newborn infants, found reduced digit ratios in both hands of male infants regardless of whether the infants were inbred or outbred. In their study, however, digit lengths were directly measured with Vernier callipers and not by an indirect method. A recent population study by Butovskaya, et al.^4^, involving 7582 individuals from Sub-Saharan Africa, Asia and Europe found lower 2D:4D ratios in males compared to females. In contrast with our findings, a previous study by Yamada, et al.^6^, involving 1045 children aged 1½ years from the Aichi regional sub-cohort of the Japan Environment and Children's Study (JECS-A) cohort study in Japan, found no differences in digit ratio between boys and girls. The authors adopted an easy-to-use photographic method to measure digit lengths. They explained that their findings may be due to difficulties in digit measurements at that age or the rapid growth that occurs at that stage of human development that may have neutralized the in-utero sexual dimorphism^6^. A similar study by Barrett, et al.^21^ among 321 children from the Infant Development and the Environment Study (TIDES) in America found no significant differences in digit ratios between boys and girls. The children in their study were, however, 4-year-olds and the researchers directly measured digit lengths with Vernier dial callipers. They explained that sex differences in digit ratios in children, tended to be less consistent and the effect sizes, smaller compared to adults. They also suggested that method variation in digit measurements may have accounted for the inconsistencies in the results of previous studies. There is substantial evidence supporting the sexual dimorphic nature of the 2D:4D ratios in humans, However, there are significant within and between populations variabilities which may be due differences in genetic and environmental factors^1^.

The average digit ratios in this study were < 1.00. Previous studies on digit ratios have been carried out among Black Africans and Afro-Caribbean populations. Consistent with this study, low 2D:4D ratios has been found among Jamaican children and their mothers^19,22,23^. Also, studies in Sub-Saharan Africa found a mean digit ratio < 1.0 among females including ethnic groups such as the Andoni (Obolo)^24^, the Igbos^25,26^, the Ebira^27,28^, the Yoruba^25^, and the Hausa^29^, all of Nigeria, Datoga and Meru of Tanzania^30^ and the Zulus of South Africa^31^. However, one study among the Himba tribe of Namibia reported an average digit ratio ≥ 1.0^32^. Reduced digit ratios among West African populations may be resulting from high rates of polygyny. Ghana and other West African countries were at the heart of the African diaspora and there is much that is similar between these countries and Jamaican populations and this includes similarities in genetics and socio-cultural variables^19,33^. Although digit ratios of the mothers and children were < 1.00 on average, mothers’ digit ratios were significantly higher than their sons and daughters in both hands. This was consistent with the findings in a study by Ventura, et al.^7^. A previous mother-daughter study in Liverpool revealed slightly higher digit ratios in mothers compared to daughters although the difference was not statistically significant.

Our study showed that mothers showed rightward bias while their sons and daughters exhibited leftward bias. A previous study by Richards, et al.^34^ found that 2D:4DL was considerably higher than 2D:4DR in boys but found no differences in girls. This study used data from a previous study by Ventura, et al.^7^ where digit ratios were estimated from photocopies of 106 newborn babies. Another study involving 1013 participants from 4 countries, found asymmetry in digit lengths. The age range in this study was, however, between 2 and 90 years and digit lengths were directly measured using Vernier callipers. Voracek, et al.^11^, studied digit ratios in a sample of about 3000 participants and found that leftward bias was more frequent in males while rightward bias was more frequent in females. The study by Voracek, et al.^35^, was a replication of the findings of a previous study by Puts et al.^36^ who analysed data from about 500 individuals. Similar studies among Black Africans have reported rightward bias among females^25,28,37^ but others reported leftward bias^38,39^ while some found no asymmetry in digit ratios^26,27,29,40^. Differences in directional asymmetry are a further indication of the effect of prenatal androgen exposure, genetics and environment and their influences on digit ratios^19^.

There were considerable negative correlations between age and the 2D:4DL in both sons and daughters in this study. This was in contrast with the findings of Manning^1^ who found no significant correlation between digit ratios and age among 800 individuals from Merseyside. However, his study involved males and females aged between 2 and 25 years. According to Manning, this was an unusual feature for a sexually dimorphic trait. But, Körner et al.^41^ who also used scanned images in a cohort study involving 274 children in Düsseldorf, Germany and measured digit ratios in both hands of males and females at ages 5, 9, 20 and 40 months, reported that age had an effect on digit ratios from ages 20–40 months. Their study population was, however, predominantly Caucasian. Manning, et al.^8^ earlier hypothesized that digit ratios were established in utero and only gain stability about 2 years of age after the postnatal testosterone surge. However, in his later research among Jamaican children, he observed a significant relationship between digit ratios measured from X-rays images, and those from photocopies, taken 2.5 years later and concluded that the 2D:4D ratio showed stability among children across periods of rapid growth^1^. In contrast to Manning, et al.^8^, previous longitudinal studies, one from the Jamaican Symmetry Project, have reported that 2D:4D digit ratios increased from infancy to adulthood^9,10^. A 2D:4D digit ratio associations studies in Wales by Richards et al.^42^ found a positive correlation between age and digit ratios in children but a negative correlation in adults. In their study, however, digit lengths were measured from hand scans in 585 parent–child pairs who were mostly White European, aged from 5 to 89 years. A recent intercontinental study involving 7582 individuals encompassing Europeans, Asians and Sub-Saharan Africans have demonstrated a positive correlation between digit ratios and age^4^.

Correlational analysis in this study found a positive relationship between mother and child but only in 2D:4DR. In line with our findings, a study involving 673 mother–child pairs, whose hands scans were studied for 2D:4D found positive associations between mother and child digit ratios. However, this study involved children who were 2–5-year-olds from the MIREC (Maternal-Infant Research on Environmental Chemicals) cohort study in Canada^12^. Also, a study among families of the Chuvasha and Bashkortostan Autonomies of the Russian Federation revealed parent–offspring correlations in 2D:4D digit ratios. Instead of hand scans, the authors' measured digit ratios from X-ray radiographic images in 1541 individuals^13^. A positive correlation between the 2D:4D digit ratio of mothers and newborn daughters have been reported by Ventura et al.^7^ and Richards et al.^42^. Similar studies among Afro-Caribbean mother–child populations have reported significant correlations in digit ratios^1^; These studies include correlation between maternal mean right and left 2D:4D with that of children in a sample of 88 Mother–child pairs (r = 0.34, p = 0.001), and 43 mother-son pairs, (r = 0.43, p = 0.003). Previous familial studies on digit ratios have indicated that the heritability of the 2D:4D digit ratio was up to 57% for the 2D:4DR and 48% for the 2D:4DL^14^. Further evidence for the heritability of digit ratio came from Marshall who studied 64 mother–child and 41 father-child pairs in the Liverpool area of the United Kingdom as reported by Manning^1^. Although the process of digit ratio heritability is not well understood and will require further studies, genetic factors have been suggested^12,14^.

This current study has many strengths. Firstly, this study is among the very few studies on digit ratios and their relationships in a mother–child population to come from Ghana and maybe the first among children under 2 years of age. Also, digit ratios were measured using a computer-assisted method which has been shown to increase measurement accuracy^43–45^. Despite the many strengths of this study, the authors acknowledge that digit ratios show within and between population variabilities which do not allow for the generalization of the results.

## Conclusion

We conclude that the 2D:4DR was considerably higher in mothers as compared to their sons and daughters while the 2D:4DR of sons was markedly lower as compared to daughters. Also, digit ratios showed rightward asymmetry in mothers, but leftward asymmetry in their sons and daughters. The left digit ratios of sons and daughters were negatively correlated with age while the right digit ratios were positively correlated with the right digit ratio of their mothers. We recommend population-specific studies since digit ratios and their relationships are influenced by genetic and environmental factors.

## Data Availability

Available on request.
